# Demonstration of novel gain-of-function mutations of αIIbβ3: association with macrothrombocytopenia and glanzmann thrombasthenia-like phenotype

**DOI:** 10.1002/mgg3.9

**Published:** 2013-04-22

**Authors:** Hirokazu Kashiwagi, Shinji Kunishima, Kazunobu Kiyomizu, Yoshiro Amano, Hiroyuki Shimada, Masashi Morishita, Yuzuru Kanakura, Yoshiaki Tomiyama

**Affiliations:** 1 Department of Hematology and Oncology, Osaka University Graduate School of MedicineSuita, Osaka, Japan; 2 Department of Advanced Diagnosis, Clinical Research Center, National Hospital Organization Nagoya Medical CenterNagoya, Aichi, Japan; 3 Department of Pediatrics, Nagano Red Cross HospitalNagano, Nagano, Japan; 4 Department of Pediatrics, Keio University School of MedicineShinjuku-ku, Tokyo, Japan; 5 Department of Pediatrics, Tosei General HospitalSeto, Aichi, Japan; 6 Department of Blood Transfusion, Osaka University HospitalSuita, Osaka, Japan

**Keywords:** Congenital macrothrombocytopenia, gain-of-function mutations, glanzmann thrombasthenia, integrin αIIbβ3, platelets

## Abstract

Integrin αIIbβ3 is indispensable for normal hemostasis, but its role for thrombopoiesis is still controversial. Recently, αIIb and β3 mutations have been identified in patients with congenital macrothrombocytopenia. We analyzed three unrelated Japanese families with congenital macrothrombocytopenia. Expression and activation state of αIIbβ3 in platelets was examined by flow cytometry and immunoblotting. Sequence of whole coding region and exon–intron boundaries of *ITGA2B* and *ITGB3* genes was performed. The effects of mutations on αIIbβ3 activation state and phosphorylation of FAK were analyzed in transfected cells. We newly identified three mutations: two mutations in highly conserved Gly-Phe-Phe-Lys-Arg sequence in juxtamembrane region of αIIb, p.Gly991Cys and p.Phe993del, and one donor site mutation of intron 13 of *ITGB3* leading to 40 amino acids deletion, p.(Asp621_Glu660del), in the membrane proximal β-tail domain of β3. One patient, who showed Glanzmann thrombasthenia-like marked reduction in surface αIIbβ3 expression (3–11% of normal control), was a compound heterozygote with *ITGA2B* p.Gly991Cys and a novel nonsense mutation, *ITGA2B* p.Arg422*. All three mutations, *ITGA2B* p.Gly991Cys, *ITGA2B* p.Phe993del, and *ITGB3* p.(Asp621_Glu660del), led to highly activated conformation of αIIbβ3 and spontaneous tyrosine phosphorylation of FAK in transfected cells. These results suggest that gain-of-function mutations around membrane region of αIIbβ3 lead to abnormal platelet number and morphology with impaired surface αIIbβ3 expression.

## Introduction

Integrin αIIbβ3 plays essential roles in thrombosis and hemostasis as a platelet receptor for fibrinogen and von Willebrand factor, but its role for normal platelet production and morphology is still controversial. Glanzmann thrombasthenia (GT) is a congenital bleeding disorder due to qualitative or quantitative defects of αIIbβ3, and platelet counts and morphology in GT are usually normal (Tomiyama 2000; Nurden et al. 2011a). Slight but not statistically significant decrease of platelet number with normal morphology was reported in β3-knockout mice (Hodivala-Dilke et al. 1999), whereas abnormalities in platelet counts and morphology have not been reported in αIIb-knockout mice or αIIbβ3-deficient dogs (Lipscomb et al. 2000; Tronik-Le Roux et al. 2000). However, Larson and Watson (2006) showed that αIIbβ3 and fibrinogen interaction regulates proplatelet formation in mice. Several αIIbβ3 mutations have been identified in patients with congenital macrothrombocytopenia: p.Asp723His in β3 (Ghevaert et al. 2008), deletion of p.(Asp621_Glu660) in β3 (Gresele et al. 2009), p.Arg995Gln and p.Arg995Trp in αIIb (Peyruchaud et al. 1998; Kunishima et al. 2011). These findings suggest involvement of αIIbβ3 on normal platelet production. Interestingly, all of αIIbβ3 mutations reported in congenital macrothrombocytopenia so far are located in membrane proximal region of αIIb or β3 and caused conformational changes which induce spontaneous binding of an activation-dependent ligand-mimetic antibody, PAC1, when they were expressed in transfected cells (Peyruchaud et al. 1998; Ghevaert et al. 2008; Kunishima et al. 2011) or megakaryocytes obtained from the patient (Bury et al. 2012). Moreover, GT-like marked reduction in surface expression levels of αIIbβ3 was reported in a homozygote of gain-of-function mutation, *ITGB3* p.Cys560Arg, and a compound heterozygote of *ITGA2B* p.Arg995Gln with a splicing acceptor site mutation in *ITGA2B* which led to absence of αIIb expression (Ruiz et al. 2001; Nurden et al. 2011b).

In this report, we demonstrate three novel αIIbβ3 mutations associated with congenital macrothrombocytopenia: two mutations, *ITGA2B* p.Gly991Cys and *ITGA2B* p.Phe993del, in highly conserved Gly-Phe-Phe-Lys-Arg sequence in juxtamembrane region of αIIb, and one mutation in the donor site of intron 13 of *ITGB3*, c.2134+1G>A, which was at the same position reported by Gresele et al. (2009) (c.2134+1G>C), leading to p.(Asp621_Glu660del) in β3. All these mutations lead to spontaneous activation of αIIbβ3 in transfected cells. We also demonstrate one patient who showed compound heterozygote with *ITGA2B* p.Gly991Cys and a novel nonsense mutation in *ITGA2B* p.Arg422*, leading to GT-like marked reduction in surface αIIbβ3 expression levels associated with macrothrombocytopenia.

## Patients, Materials, and Methods

### Cases

**Figure 1:**
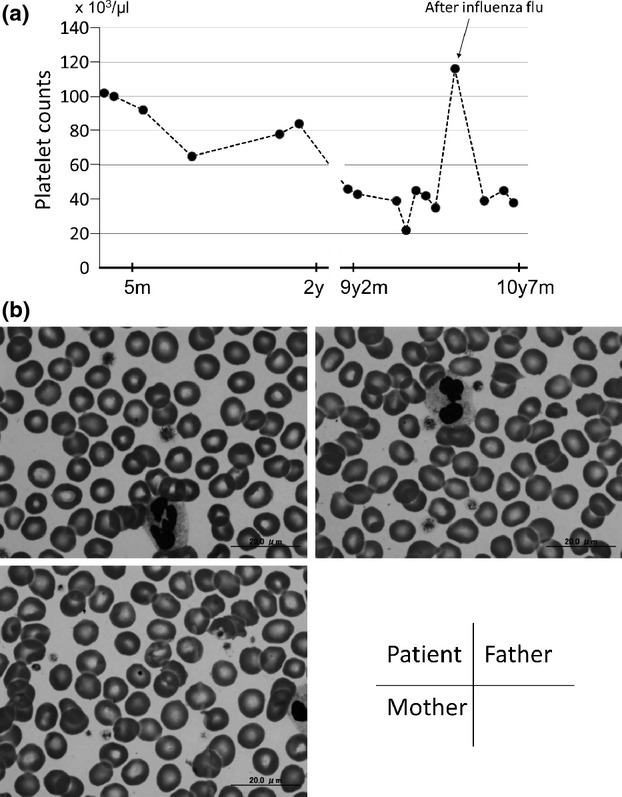
Transition of platelet counts of case 1 (a) and morphology of platelets of case 1 family members (b).

The first case is a 9-year-old Japanese girl. She had a history of purpura with platelet counts of 60–100 × 10^3^/μL from the birth to 2 years of age. Since her bleeding diathesis with easy epistaxis, bruising, and hemostatic difficulty after teeth extraction became worse at 9 years of age, she was referred to our hospital. Mild thrombocytopenia was reported in her father and a paternal aunt. Hematological examination at our hospital revealed that platelet counts of the propositus were around 40 × 10^3^/μL with increase of mean platelet volume (MPV) except for transient increase in platelet counts after influenza flu infection (Fig. 1a, Table 1). Increase of platelet size was confirmed under microscope (Fig. 1b, Table 1). Percentages of reticulated platelets were 9.1–9.5% (normal range: 1.4–9.1%) and plasma thrombopoietin levels were 4.1–6.0 pg/mL (normal range: <106 pg/mL). Platelet counts of her father were 70–109 × 10^3^/μL with slight increase of MPV and platelet size, whereas platelet counts, MPV, and platelet size were within normal range in her mother (Fig. 1b, Table 1).

**Table 1:** Platelet characteristics, mutations and bleeding tendency of cases

	Platelet count × 10^3^/μL	MPV fL (7.2–9.7)	Platelet size μm (2.5 ± 0.3)	αIIbβ3 (%control)	CD42b (%control)	Mutations	Bleeding tendency
Family 1							
Father	70–109	10.0–11.7	3.1 ± 0.7	67–76	142	*ITGA2B* (p.Gly991Cys) hetero	–
Mother	179–267	7.2–8.5	2.8 ± 0.7	65–92	96	*ITGA2B* (p.Arg422*) hetero	–
Case 1	22–102	11.0–14.8	3.4 ± 0.8	3–11	181	*ITGA2B* (p.Gly991Cys)/(p.Arg422*)	purpura, epistaxis, bruising, etc
Family 2							
Mother	94	12.8	4.0 ± 1.2	75–82	130	*ITGA2B* (p.Phe993del) hetero	–
Case 2	59–111	11.9	3.4 ± 1.2	74–78	138	*ITGA2B* (p.Phe993del) hetero	–
Family 3							
Grandfather	87	n.d.	4.2 ± 1.0	65	150	*ITGB3* p.(Asp621_Glu660del) hetero	–
Mother	50–60	n.d.	5.3 ± 1.3	66	142	*ITGB3* p.(Asp621_Glu660del) hetero	hypermenorrhea
Case 3	29–113	n.d.	5.1 ± 1.0	67	132	*ITGB3* p.(Asp621_Glu660del) hetero	hematoma, bruising, etc

The second case is a 9-year-old Japanese boy. He was referred to our hospital because of bronchial asthma. Macrothrombocytopenia was pointed out by routine blood tests. He has no episode of bleeding tendency. His platelet counts were 59–110 × 10^3^/μL with increase of MPV and platelet size (Table 1). His father's and mother's platelet counts were 242 and 94 × 10^3^/μL, respectively, and mild thrombocytopenia was also reported in the maternal grandmother without bleeding tendency.

The third case is an 8-year-old Japanese girl. Her platelet count was 75 × 10^3^/μL at birth. She suffered subaponeurotic hematoma at 4 years of age without obvious trauma. Her platelet count was decreased to 29 × 10^3^/μL at that time and platelet transfusion was performed. Because thrombocytopenia around 50–100 × 10^3^/μL had been persisted since then, she was referred to our hospital. Her mother had bleeding diathesis with lifelong easy bruising and a history of difficulty in hemostasis at appendectomy. Thrombocytopenia with 50–60 × 10^3^/μL was pointed out in her first pregnancy. Mild thrombo-cytopenia was also noticed in grandfather of the propositus without bleeding tendency. Increase of platelet size was observed in the propositus, her mother and grandfather (Table 1).

In all cases, *MYH9* disorders, heterozygous and homozygous Bernard-Soulier syndrome, and type 2B von Willebrand disease were excluded by their phenotypes, that is, morphology of platelets and white blood cells, the expression levels of GPIb (CD42b), and activity of von Willebrand factor, respectively.

### Reagents

For detection of αIIbβ3 expression, IOP41a (αIIb; Immunotech, Marseille, France), 5B12 (αIIb; Dako Denmark, Glostrup, Denmark), P2 (αIIbβ3; Beckman Coulter Japan, Tokyo, Japan), VIPL3 (αIIbβ3; Becton Dickinson, BD, Franklin Lakes, NJ), VIPL2 (β3; BD), and SZ21 (β3; Beckman Coulter) were used. For detection of GPIb expression, HIP1 (BD) and SZ2 (Beckman Coulter) were used. Rabbit polyclonal anti-αIIbβ3 antibody and PT25-2 were generous gifts from Dr. T. J. Kunicki (The Scripps Research Institute, La Jolla, CA) and Dr. M. Handa (Keio University, Tokyo, Japan), respectively.

### Platelet glycoprotein expression and functional assay

Blood samples were obtained after written informed consent from all patients or family members in accordance with the Declaration of Helsinki. Institutional Review boards of Osaka University Hospital and each of the participating institutions and hospitals approved this study. All experiments were performed within 48 h after blood sampling. Platelet glycoprotein expression was analyzed by flow cytometry and Western blotting as previously described (Kiyoi et al. 2003). Activation state of αIIbβ3 was assessed by binding of the ligand-mimetic activation-dependent antibody, PAC1 (BD), and expressed as activation index, defined as (*F*_x_−*F*_min_)/(*F*_max_−*F*_min_). *F*_x_ is the mean fluorescent intensity (MFI) of PAC1 binding to the resting platelets. *F*_min_ and *F*_max_ are MFIs of PAC1 binding in the presence of a Arg-Gly-Asp mimetic antagonist, FK633, to resting platelets and MFIs of PAC1 binding to phorbol-12-myristate-13-acetate (PMA)-activated platelets, respectively.

### Platelet morphology

Peripheral blood smears were stained with May-Grünwald Giemsa (original magnification 1000×). Images were obtained using a BX51 microscope with a 100x/1.35 numeric aperture oil objective (Olympus, Tokyo, Japan). Images of the slides were acquired using a DP71 digital camera and DP Controller software Version 3.1.1.267 (Olympus). Images were converted to gray scale mode and contrast and brightness were adjusted with Adobe Photoshop CS5 Version 12.0 (Adobe Systems, San Jose, CA).

### Genetic analysis

The entire coding sequence of exons and exon–intron boundaries of *ITGA2B* and *ITGB3* and the whole coding regions of *ITGA2B* and *ITGB3* cDNA obtained from platelets were sequenced as previously described (Kiyoi et al. 2003; Kunishima et al. 2011).

In case of *ITGA2B* p.Phe993del, PCR products obtained from amplification of exon 30 of *ITGA2B* from the patient's DNA were subcloned to pCR2.1-TOPO vector (Life technologies, Carlsbad, CA) and sequence analysis was performed.

### Mutagenesis and transfection assay

*ITGA2B* p.Gly991Cys and *ITGA2B* p.Phe993del mutations were introduced to full-length *ITGA2B* cDNA in pcDNA3 vectors by a site-directed mutagenesis kit according to the manufacturer's instructions (Agilent Technologies, Santa Clara, CA). Full-length of *ITGB3* cDNA having p.(Asp621_Glu660del) was obtained by RT-PCR amplification from the patient's platelet mRNA and cloned into pcDNA3 vector. Transient transfection of αIIbβ3 mutant vectors to 293T cells were performed as described previously and activation state of expressed αIIbβ3 was assessed by PAC1 binding (Kashiwagi et al. 1999; Kiyomizu et al. 2012). In brief, cells were incubated with FITC-PAC1 and PE-CD61 (BD) with or without FK633 or an αIIbβ3 activating antibody, PT25-2. PAC1 bindings to CD61-high expressing cells were assessed and activation index was calculated as done in platelets except that *F*_max_ was MFI of PAC1 binding in the presence of PT25-2.

Focal adhesion kinase (FAK) phosphorylation of the transfected cells was detected as previously described (Kunishima et al. 2011). In brief, αIIbβ3-transfected 293T cells in suspension or adherent cells on 100 μg/mL fibrinogen-coated plates were lysed with 1% Triton X-100. FAK was immunoprecipitated from equal amount of lysates with anti-FAK antibody, A17 (Santa Cruz Biotechnology, Santa Cruz, CA), and phosphotyrosine was detected with 4G10 (Millipore Corp., Billerica, MA). To monitor the loading of gel lanes, the membrane was stripped and reprobed with A17.

### Statistics

Statistical significance was evaluated by two-tailed paired Student's *t*-test. *P*-value which is <0.05 was considered as significant.

## Results

### Case 1: Compound heterozygote of *ITGA2B* p.Gly991Cys and *ITGA2B* p.Arg422* with GT-like phenotype

**Figure 2:**
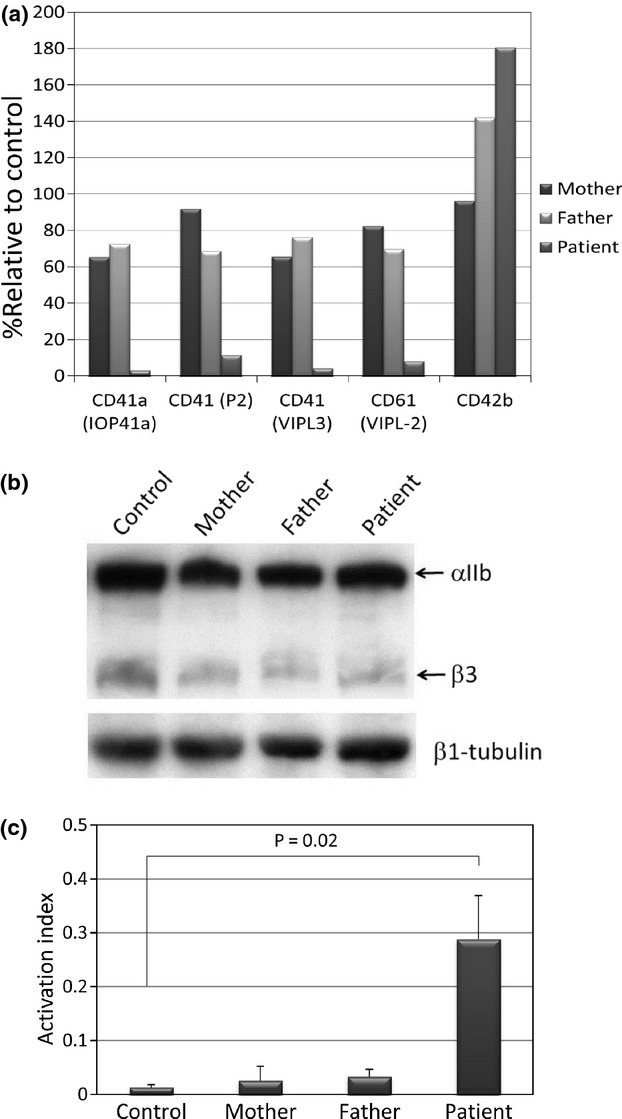
Expression of αIIbβ3 in case 1 family members. (a) Surface expression of αIIbβ3 and CD42b was evaluated by binding of indicated monoclonal antibodies in flow cytometry. Percent relative binding against normal control was demonstrated. Shown are representative results of three independent experiments. (b) Total expression of αIIbβ3 was evaluated by Western blotting using rabbit polyclonal anti-αIIbβ3 antibodies. Protein loading of each well was assessed by monoclonal anti-β1-tubulin antibody (Sigma). Shown are representative results of three independent experiments. (c) Activation index of αIIbβ3. Relative PAC1 binding in the resting platelets compared with maximal PAC1 binding in the platelets stimulated with PMA is defined as activation index. Shown are means and standard deviations of three independent experiments.

Flow cytometric analysis showed that surface αIIbβ3 expression in platelets of case 1 was markedly impaired (3–11% of control), and was mild∼moderately decreased in platelets of her father (67–76%) and mother (65–92%). These phenotypes of case 1 platelets were consistent with type II GT except for macrothrombocytopenia (Fig. 2a, Table 1). Total αIIbβ3 expression in platelet lysates examined by Western blotting using anti-αIIbβ3 polyclonal antibodies showed that the patient platelets contained ∼60% amount of αIIbβ3 compared with normal control platelets (Fig. 2b). There was no apparent increase in MFI of PAC1 binding to the patient's platelets compared with the control platelets at the resting state; 4.31 ± 0.76 for case 1 versus 4.30 ± 0.85 for the control (mean ± SD, *n* = 3) (Fig. S1A). However, the activation index, which represents relative PAC1 binding to the resting platelets against maximal PAC1 binding to the PMA-stimulated platelets, suggested that the patient's platelets expressed constitutive active αIIbβ3 (Fig. 2c). MFI of PAC1 binding to father's platelet (6.46 ± 1.45) was slightly but significantly increased at the resting state compared with the control platelets (*P* = 0.03) (Fig. S1A), and the activation index was also increased although it was not statistically significant (0.033 ± 0.013 for father versus 0.013 ± 0.005 for the control, *P* = 0.07) (Fig. 2c). ADP-induced *α*-granule secretion assessed by CD62P expression appeared to be impaired in the patient's platelets and the impairment was statistically significant in 10 μmol/L ADP stimulation compared with the control platelets (*P* = 0.03; Fig. S1B).

**Figure 3:**
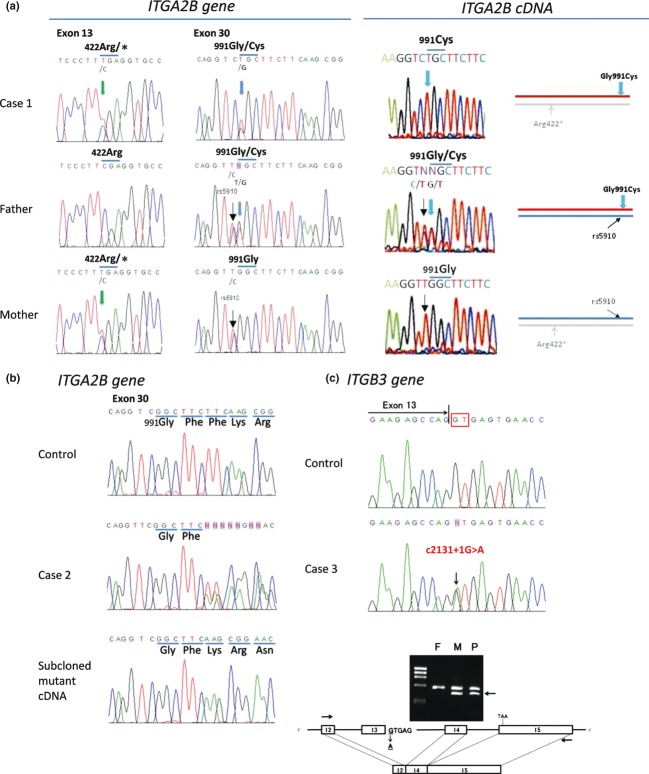
Genetic analysis of three cases. (a) Sequences of *ITGA2B* gene are shown in left two panels, and sequences and schematic representation of platelet *ITGA2B* cDNA are shown in right panels. *ITGA2B* gene of case 1 had two mutations, a C to T substitution in exon 13, leading to p.Arg422* (left panels), and a G to T substitution in exon 30, leading to p.Gly991Cys mutation (mid-left panels). *ITGA2B* gene of father had p.Gly991Cys mutation, and that of mother had p.Arg422* mutation. Note that both father and mother had a common SNIP, rs5910: (C>T), just adjacent the G to T substitution in exon 30. Only transcripts of the p.Cys991 allele derived from father (red lines in the right panels) were detected in platelet *ITGA2B* cDNA of case 1 (mid-right panel), indicating that the amounts of transcripts of the p.Arg422* allele derived from mother (light gray lines in the right panels) were much decreased. (b) *ITGA2B* gene of case 2 showed mixed sequence after Phe992 in exon 30 (mid panel). Sequence of the mutant clone after cloning of the PCR products showed that three nucleotides, TTC, were deleted, leading to a deletion of p.Phe993 (lower panel). (c) *ITGB3* gene of case 3 showed a G to A mutation, c.2134+1G>A, in the acceptor site of exon 13 (mid panel). RT-PCR analysis of *ITGB3* mRNA between exon 12 and 15 obtained from case 3 (P) and her parent (F, M) platelets showed that *ITGB3* mRNA of case 3 and her mother had both normal-sized mRNA and 120 bp-deleted mRNA (arrow), which is corresponding to exon 13 skipping (lower panel).

Sequence analysis of entire coding regions of exons and exon–intron boundaries of *ITGA2B* and *ITGB3* gene of the patient revealed two novel mutations in *ITGA2B*; C to T substitution in exon 13 which makes stop codon at arginine-422 (p.Arg422*), and G to T substitution in exon 30 which leads to substitution of glycine-991 to cysteine (p.Gly991Cys) (Fig. 3a). Sequence of her parents' DNAs indicated that *ITGA2B* p.Arg422* was derived from her mother and *ITGA2B* p.Gly991Cys was derived from her father. Analysis of platelet *ITGA2B* mRNA indicated that transcripts of *ITGA2B* p.Arg422* allele were very low, as we could detect only *ITGA2B* p.Gly991Cys mRNA in the patient's platelets (Fig. 3a).

PolyPhen-2, a software for predicting damaging effects of missense mutations, predicted that p.Gly991Cys mutation was probably damaging to the protein function with a score of 1.000 (sensitivity: 0.00; specificity: 1.00) (Adzhubei et al. 2010).

### Case 2: Heterozygote of *ITGA2B* p.Phe993del

Surface expression of αIIbβ3 in platelets of the patient and his mother was around 80% compared with control subjects with increase of CD42b expression (Table 1). MFIs of PAC1 binging to the resting platelets of the patient and mother were essentially the same as to the control platelets, but the activation indexes were slightly increased in the patient (0.032) and mother (0.022) compared with the control (0.015) (Fig. S2A). Platelet spreading on immobilized fibrinogen shows high heterogeneity of platelet size of the patient (Fig. S2B).

Sequence analysis revealed that the patient and his mother were heterozygous of a novel in-frame three nucleotides, TTC, deletion in exon 30 of *ITGA2B*, leading to a deletion of phenylalanine-993 in αIIb (Fig. 3b).

### Case 3: Heterozygote of *ITGB3* p.(Asp621_Glu660del)

Surface expression levels of αIIbβ3 in platelets of the patient, her mother, and grandfather were decreased to around 65% of normal subjects with increase of CD42b expression (Table 1). Western blotting using anti-β3 antibodies indicated that platelets of the patient contained normal β3 and a low-molecular-weight β3 (Fig. S3A). Spontaneous binding of PAC1 was modestly observed in platelets of the patient (Fig. S3B).

Sequence analysis of DNA revealed the patient was a heterozygote of a novel G to A substitution in the donor site of intron 13 of *ITGB3*, c2143 + 1G > A. RT-PCR assay around exon 13 of *ITG3B* revealed that both the patient and her mother had normal-sized and ∼200 bp short *ITGB3* cDNA (Fig. 3c). We confirmed deletion of exon13 in the short cDNA by sequencing of RT-PCR products. The same mutation was also detected in her grandmother (data not shown).

### Expression assay

**Figure 4:**
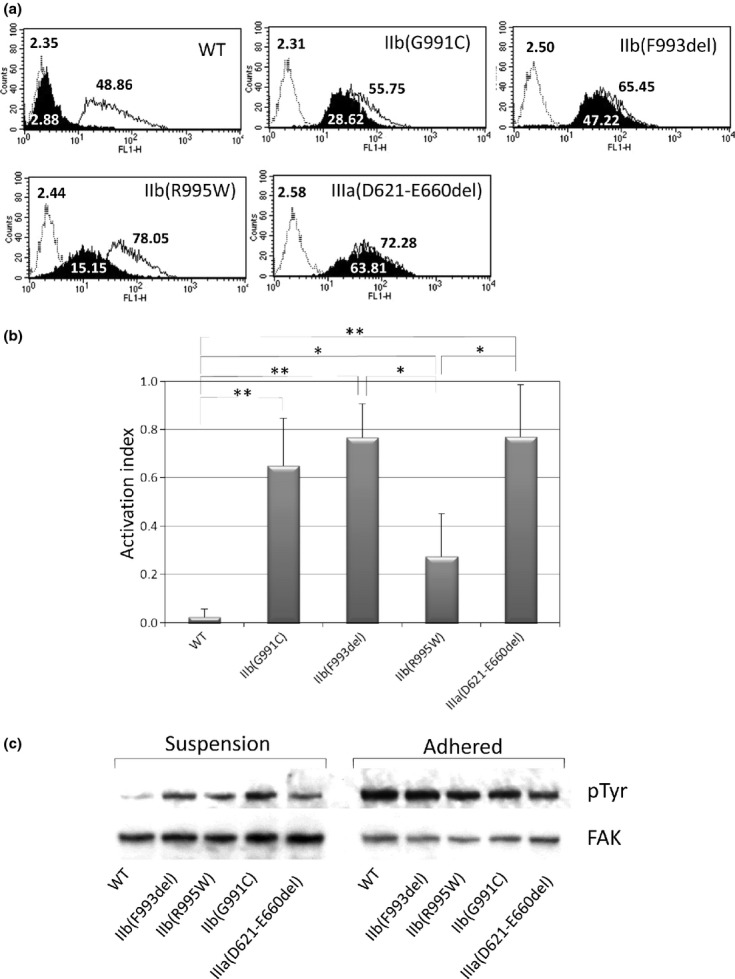
Expression assay of mutant αIIbβ3. (a) PAC1 bindings to CD61 highly expressed cells in the resting state (shaded), with FK633 (dotted lines), and with PT25-2 (solid lines) are shown. Mean fluorescence intensity of each condition is also indicated. Shown are representative results of three independent experiments. (b) Relative PAC1 binding compared with PAC1 binding in the presence of PT25-2 to each mutant was defined as activation index. Shown are means and standard deviations of three independent experiments. *P < 0.05. **P < 0.01 (c) Tyrosine phosphorylation of FAK was detected with antiphosphotyrosine antibody, 4G10, after immunoprecipitation of FAK with anti-FAK antibody, A17 (upper). Equal amount of gel loading was monitored by blotting with A17 (lower). Note that 400 μg and 300 μg lysates from suspension and adhered cells on fibrinogen-coated plates, respectively, were used for immunoprecipitation. Shown are representative results of three independent experiments.

The data obtained from patients' platelets suggested that the novel mutations lead to activation of αIIbβ3 like other αIIbβ3 mutations associated with macrothrombocytopenia. To examine the effect of each mutation on activation state of αIIbβ3 precisely, we assessed PAC1 binding to mutated αIIbβ3 expressed in 293T cells. Spontaneous binding of PAC1 was observed in all three mutants, αIIb(Gly991Cys)β3, αIIb(Phe993del)β3, and αIIbβ3(Asp621_Glu660del), and the activation states of these mutants appeared to be higher than that of αIIb(Arg995Trp)β3 (Fig. 4a and b).

We and others previously observed spontaneous phosphorylation of FAK in αIIb(Arg995Trp)β3- or αIIbβ3(Asp621_Glu660del)-expressing cells (Kunishima et al. 2011; Bury et al. 2012). In addition to αIIb(Arg995Trp)β3- or αIIbβ3(Asp621_Glu660del)-transfected cells, FAK in αIIb(Phe993del)β3 or αIIb(Gly991Cys)β3-transfected cells were also phosphorylated even under suspension conditions (Fig. 4c).

## Discussion

In this report, we described three novel αIIbβ3 mutations detected in three unrelated Japanese families with congenital macrothrombocytopenia. Two mutations, p.Gly991Cys and p.Phe993del in *ITGA2B* gene, were both in highly conserved juxtamembrane Gly-Phe-Phe-Lys-Arg sequence of αIIb. One mutation was a G to A mutation in the donor site of intron 13 of *ITGB3*, which leads to exon13 skip resulting in 40 amino acids (Asp621_Glu660) deletion in the membrane proximal β-tail domain of β3. The same exon 13 skip caused by a G to C mutation at the same position has been previously identified in Italian families with congenital macrothrombocytopenia (Gresele et al. 2009). All three mutants expressed in transfected cells led to highly activated conformation of αIIbβ3 and spontaneous activation of FAK. Furthermore, we found case 1 was a compound heterozygote of *ITGA2B* p.Gly991Cys and a novel nonsense mutation, *ITGA2B* p.Arg422*, and her platelets showed GT-like phenotype with macrothrombocytopenia.

**Figure 5:**
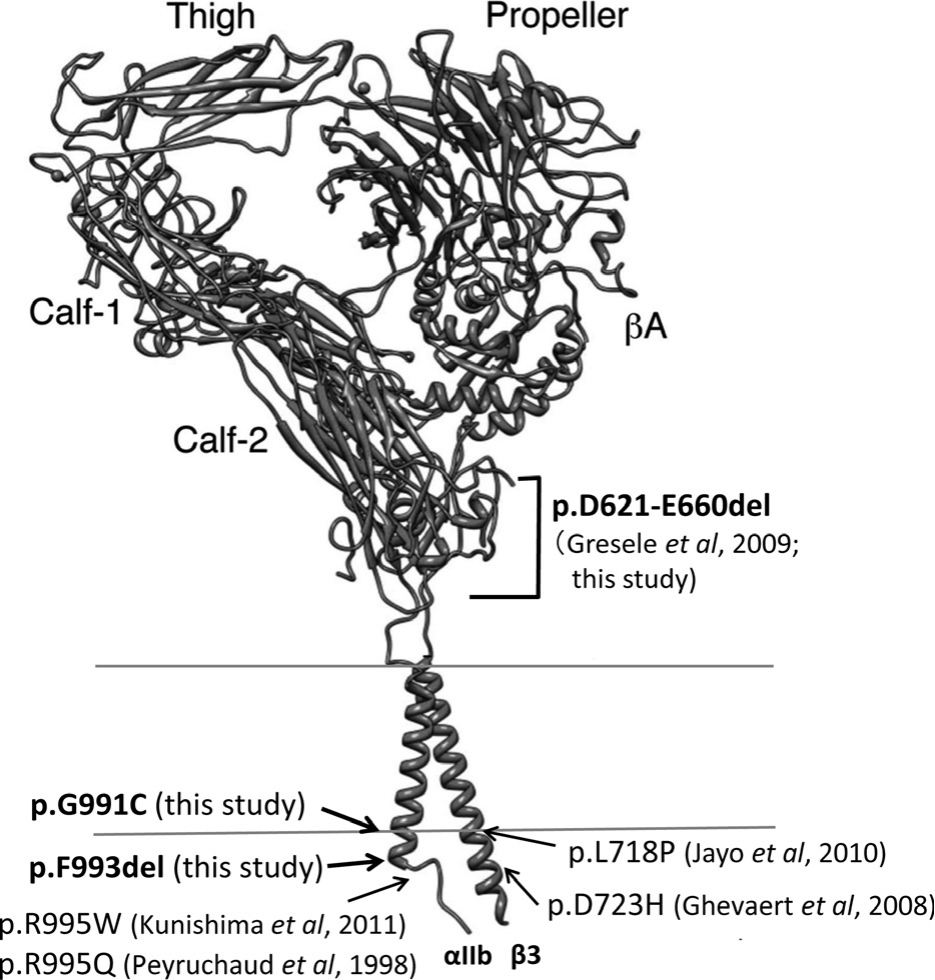
Schematic representation of the location of αIIbβ3-activating mutations associated with congenital macro-thrombocytopenia. Cristal structure of αIIbβ3 was adopted from Ulmer's paper (Ulmer 2010).

Almost 200 mutations in *ITGA2B* and *ITGB3* have been reported by extensive investigations of GT patients (the GT data base; http://sinaicentral.mssm.edu/intranet/research/glanzmann). Majority of the mutations causes a defect in αIIbβ3 biosynthesis, leading to little or no surface expression of αIIbβ3. Rare αIIbβ3 mutations cause dysfunction of its ligand-binding ability, which are referred as “variant type”. Another type of αIIbβ3 mutations, which lead to activated conformation of αIIbβ3, has been also reported. These gain-of-function mutations can be classified generally in two groups: one is located in extracellular cysteine residues in β3 and the other is located in membrane proximal regions of αIIb or β3. One of most studied gain-of-function mutation is *ITGB3* p.Cys560Arg. A homozygote of this mutation showed a mild bleeding diathesis with platelet counts of 100–150 × 10^3^/μL and GT-like reduced surface αIIbβ3 expression (∼20% of normal) (Ruiz et al. 2001). Other gain-of function mutations in cysteine residues in β3, such as p.Cys542Arg, p.Cys457Tyr, p.Cys598Tyr, and p.Cys549Arg, primarily cause highly impaired surface expression of αIIbβ3 with normal platelet counts (Nurden et al. 2011a,b). In contrast, gain-of-function mutations in membrane proximal regions have been identified in subjects with congenital macrothrombocytopenia, and three mutations reported in this paper are also located in this region (Fig. 5). These results suggest that active conformation of αIIbβ3 per se may not be essential for the morphological and quantitative abnormalities of platelets. Partial, but not full, activation of αIIbβ3 might be important as suggested by Schaffner-Reckinger et al. (2009), although αIIb(Gly991Cys)β3, αIIb(Phe993del)β3, and αIIbβ3(Asp621_Glu660del) showed almost full activation state in the transfected cells (Fig. 4a and b). We and others demonstrated that constitutive outside-in signaling was induced by gain-of-function mutations around juxtamembrane region of αIIbβ3 (Fig. 5c) (Kunishima et al. 2011; Bury et al. 2012). Schaffner-Reckinger et al. (2009) also suggested that downregulation of RhoA activity by αIIbβ3(Asp723His) induces microtubule-driven proplatelet formation in αIIbβ3(Asp723His)-transfected CHO cells. In case of *ITGB3* p.Leu718Pro, abnormal clustering of αIIbβ3 was observed in platelets and transfected cells (Jayo et al. 2010). These aberrant outside-in signaling and/or aberrant clustering of αIIbβ3 may interfere with proper megakaryopoiesis and cause macrothrombocytopenia. This hypothesis remains to be determined.

Regardless of its location, impairment of surface αIIbβ3 expression is constantly observed in gain-of-function mutations of αIIbβ3. Especially, the impairment is evident in homozygote of mutations, like the *ITGB3* p.Cys560Arg patient (Ruiz et al. 2001), or compound heterozygote with a nonsense mutation, like the *ITGA2B* p.Arg995Glu patient (Hardisty et al. 1992; Nurden et al. 2011b). Interestingly, despite the marked reduction in surface expression, cytoplasmic pool of αIIbβ3 was largely retained and recovery of surface expression of αIIbβ3 was observed after agonist stimulation in these patients (Hardisty et al. 1992; Nurden et al. 2011b). Similarly, we observed GT-like severe reduction in surface αIIbβ3 expression levels (3–11% of control) with retention of substantial amounts of αIIbβ3 (∼60%) in cytoplasmic pool of platelets in case 1, who is a compound heterozygote of *ITGA2B* p.Gly991Cys and *ITGA2B* p.Arg422*. However, we did not observe clear recovery of surface αIIbβ3 expression in the patient's platelets even with PMA or ADP stimulation (Fig. S1A). Conformational change induced by αIIb(Gly991Cys) mutation may profoundly affect transportation of αIIbβ3 on cell surface.

We observed severe decrease of platelet number of case 1 at her 9 years of age, despite that her thrombocytopenia was comparable with that of her father in her infancy, suggesting that additional acquired mechanism may contribute to her thrombocytopenia. As her menstruation had not been started and there was no severe bleeding, increased platelet consumption is unlikely. Although we did not perform bone marrow examination, normal thrombopoietin level in her plasma and increase in platelet number after flu infection suggest that profound defects of thrombopoiesis are also unlikely. Although we did not detect platelet-associated IgG and IgM antibodies or platelet-associated anti-αIIbβ3 IgG antibodies (data not shown), immunological destruction of her platelets might be involved. Long-term follow-up may be necessary to resolve the mechanism of thrombocytopenia in this case.

In summary, we described novel αIIbβ3 gain-of-function mutations associated with congenital macrothrombocytopenia. Together with our previous report (Kunishima et al. 2011), mutations in membrane proximal regions of αIIb or β3 are unexpectedly common cause of congenital macrothrombocytopenia in Japanese. Homozygosity of these mutations or compound heterozygosity with mutations leading to impairment of αIIbβ3 expression leads to GT-like phenotype with macrothrombocytopenia.

## Supporting Information

**Figure S1.** PAC1 binding (A) and CD62P expression (B) in platelets of case 1 family members. Platelets adjusted to 30 × 10^3^/μL with Tyrode's buffer were incubated with or without 10 μmol/L FK633, 200 nmol/L PMA, or the indicated concentration of ADP in the presence of FITC-PAC1 and PE-CD62P for 20 min at room temperature and then analyzed on flow cytometry. Shown are means and standard deviations of mean fluorescent intensity of three independent experiments. Statistical significance against control was evaluated by two-tailed paired Student's *t*-test. *P*-value which is <0.05 was considered as significant (*).

**Figure S2.** (A) PAC1 binding with 10 μg/mL PT25-2, 200 nmol/L PMA,or 10 μmol/L ADP to platelets of case 2 family was examined with flow cytometry. Relative percent binding against control platelets with PMA was shown. (B) Adhesion of platelets on immobilized fibrinogen. Washed platelets of case 2 were seeded on 100 μg/mL fibrinogen-coated glass coverslips and incubated for 1 h at 37°C. After gentle washing, adhered platelets were fixed with methanol and acetone, and stained with SZ22 (anti-αIIb; Beckman Coulter).

**Figure S3.** (A) Immunoblot analysis of platelet lysates obtained from case 3 (P) and control (C). αIIb was detected with SZ22 and β3 was detected with anti-β3 antibody (H-96) (Santa Cruz). Note that the patient contained normal β3 and a low-molecular-weight β3 (arrow). (B) PAC1 binding to platelets with or without RGDS peptides or 200 nmol/L PMA. Mean fluorescent intensities of each condition were indicated.
