# Supplementary material for: Demonstration of novel gain-of-function mutations of αIIbβ3: association with macrothrombocytopenia and glanzmann thrombasthenia-like phenotype
Source: Mol Genet Genomic Med. 2013 Apr 22;1(2):77–86. doi: 10.1002/mgg3.9 (PMC3865572; doi:10.1002/mgg3.9)
Supplement: Supplementary file 1 [file mgg30001-0077-SD1.pdf]

Figure S1

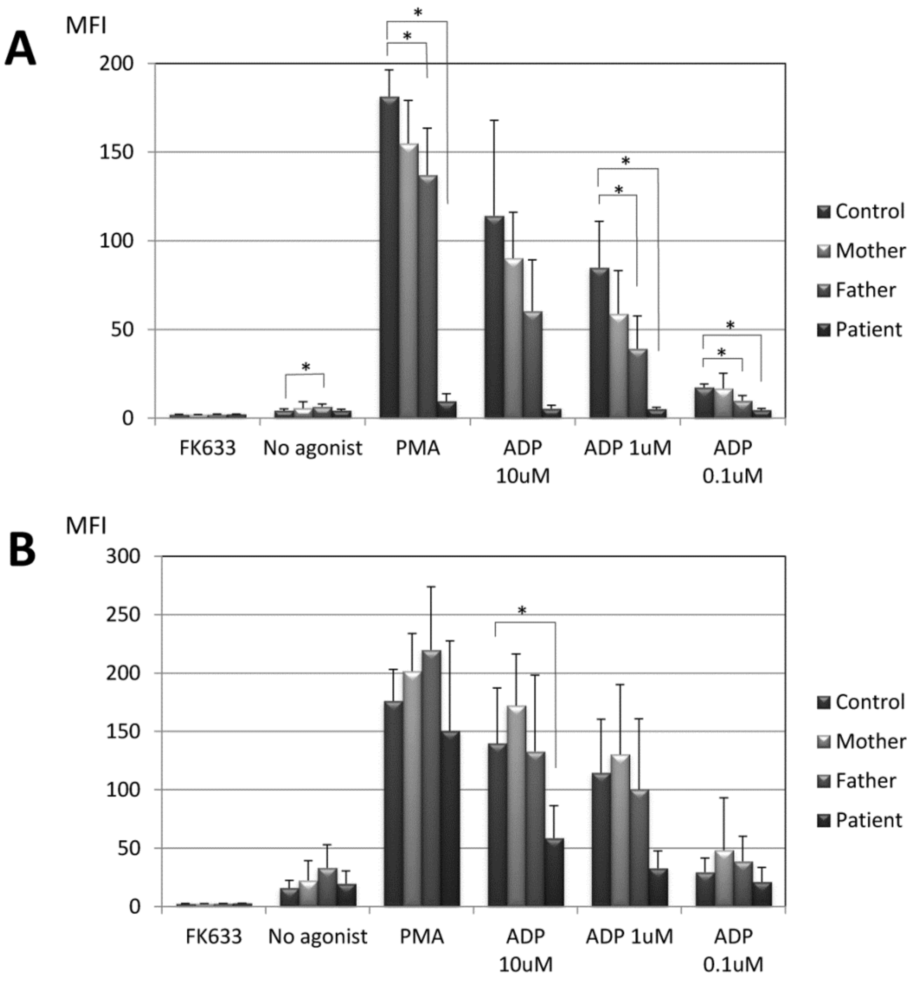

Figure S1. PAC1 binding (A) and CD62P expression (B) in platelets of case 1 family members. Platelets adjusted to 30 x 10^3/µl with Tyrode's buffer were incubated with or without 10 µM FK633, 200 nM PMA or the indicated concentration of ADP in the presence of FITC-PAC1 and PE-CD62P for 20 min at room temperature and then analyzed on flow cytometry. Shown are means and standard deviations of mean fluorescent intensity of three independent experiments. Statistical significance against control was evaluated by two-tailed paired Student's *t*-test. P-value which is less than 0.05 was considered as significant (*).

Figure S2

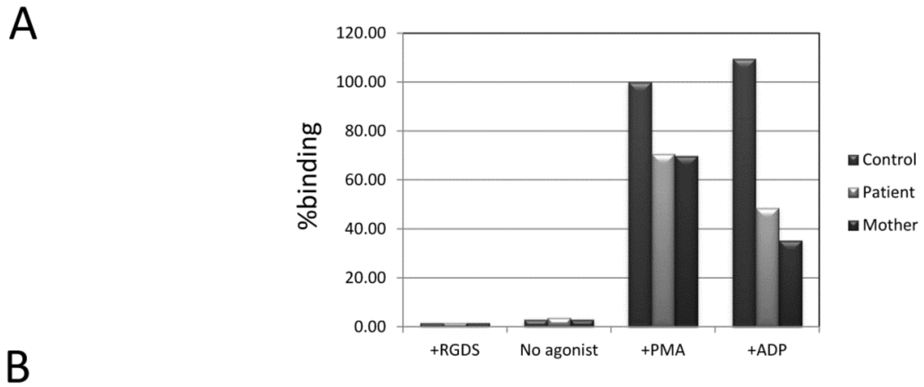

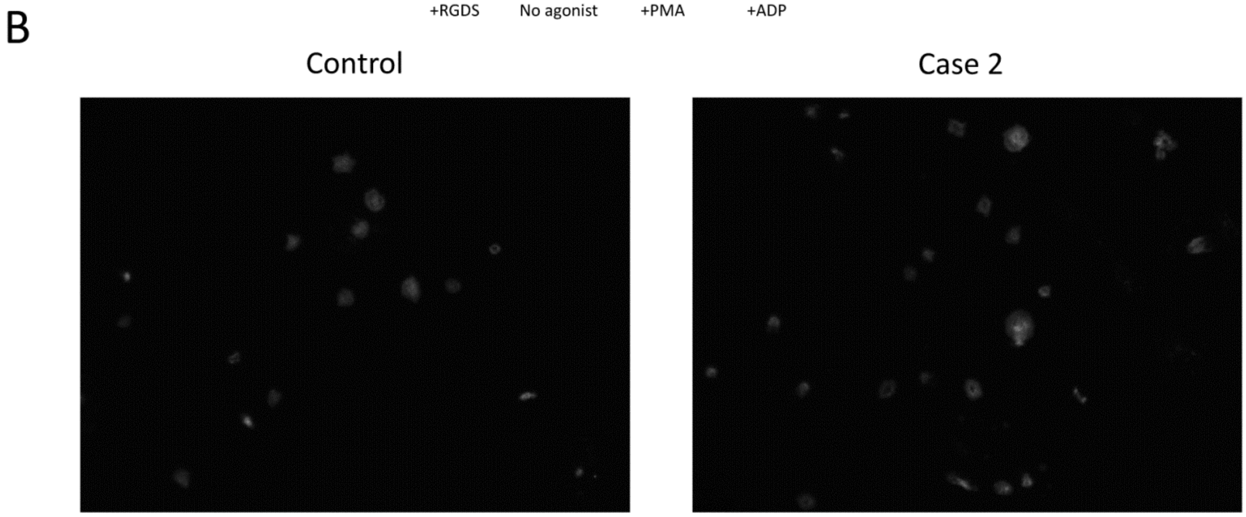

Figure S2. (A) PAC1 binding with 10 µg/ml PT25-2, 200nM PMA or 10µM ADP to platelets of case 2 family was examined with flow cytometry. Relative percent binding against control platelets with PMA was shown. (B) Adhesion of platelets on immobilized fibrinogen. Washed platelets of case 2 were seeded on 100 µg/ml fibrinogen-coated glass coverslips and incubated for 1 hour at 37°C. After gentle washing, adhered platelets were fixed with methanol and acetone, and stained with SZ22 (anti-αIIb; Beckman Coulter).

Figure S3

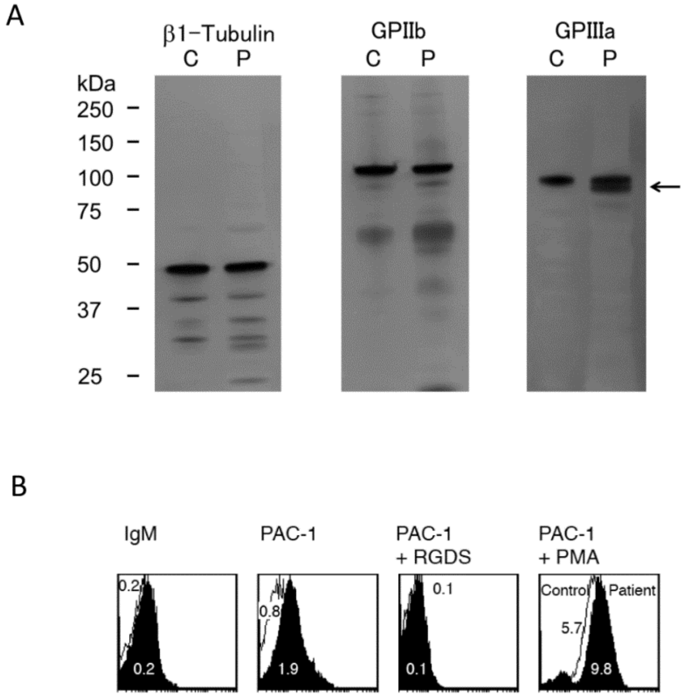

Figure S3. (A) Immunoblot analysis of platelet lysates obtained from case 3 (P) and control (C). αIIb was detected with SZ22 and β3 was detected with anti-β3 antibody (H-96) (Santa Cruz). Note that the patient contained normal β3 and a low molecular weight β3 (arrow). (B) PAC1 binding to platelets with or without RGDS peptides or 200nM PMA. Mean fluorescent intensities of each condition were indicated.
